# Investigating the Potential of Transdermal Delivery of Avanafil Using Vitamin E-TPGS Based Mixed Micelles Loaded Films

**DOI:** 10.3390/pharmaceutics13050739

**Published:** 2021-05-17

**Authors:** Abdullah A. Alamoudi, Osama A. A. Ahmed, Khalid M. El-Say

**Affiliations:** Department of Pharmaceutics, Faculty of Pharmacy, King Abdulaziz University, Jeddah 21589, Saudi Arabia; aalamoudi1@kau.edu.sa (A.A.A.); oaahmed@kau.edu.sa (O.A.A.A.)

**Keywords:** bioavailability, erectile dysfunction, transdermal film, PDE5 inhibitors, skin permeation

## Abstract

To avoid the first-pass metabolism of avanafil (AVA) and its altered absorption in the presence of food after oral administration, this study aimed to investigate the potential of TPGS-based mixed micelle (MM)-loaded film for transdermal delivery and the enhancement of bioavailability. A Box–Behnken design was employed to optimize the permeation behavior of AVA from the transdermal film across the skin. The variables were the hydrophile-lipophile balance (HLB) of the surfactant (X_1_), the concentration of mixed micelles (MMs) in the film (X_2_), and the concentration of the permeation enhancer (X_3_). The initial permeation of AVA after 1 h (Y_1_), and the cumulative permeation of AVA after 24 h (Y_2_) were the dependent variables. Ex vivo studies were carried out on freshly isolated rat skin to investigate the drug’s permeation potential and results were visualized using a fluorescence laser microscope. Moreover, the pharmacokinetic behavior after a single application on male Wistar rats, in comparison with films loaded with raw AVA, was evaluated. The results showed that the optimum factor levels were 9.4% for the HLB of the surfactant used, and 5.12% MMs and 2.99% penetration enhancer in the film. Imaging with a fluorescence laser microscope indicated the ability of the optimized film to deliver the payload to deeper skin layers. Furthermore, optimized AVA-loaded TPGS-micelles film showed a significant increase (*p* < 0.05) in the C_max_ of AVA and the area under the AVA plasma curve (approximately three-fold). The optimized AVA-loaded TPGS-MM film thus represents a successful delivery system for enhancing the bioavailability of AVA.

## 1. Introduction

Impotence/erectile dysfunction (ED) is one of the most widely recognized diseases in male sexual dysfunction, with studies showing that 1/3 of American men over age 40 are affected with ED [1,2]. Locally in Jeddah, a cross-sectional, multi-clinical study showed that more than 70% of the participants in the study showed moderate to severe ED symptoms [3]. One of the first-line therapy categories for the treatment of ED is phosphodiesterase-5 inhibitors (PDE5 inhibitors) [4,5]. PDE5 inhibitors include, for example, sildenafil, the first PDE5 inhibitor, known as Viagra^®^, which was introduced in 1998, followed later by tadalafil (Cialis^®^) and vardenafil (Levitra^®^), both of which were approved in 2003 [6]. One of the latest drugs discovered and categorized under PDE type V inhibitors is avanafil (AVA). It has been recently approved for the treatment of ED by the US FDA in 2012, marketed as Stendra^®^ by Vivus, and approved in Europe by EMA in 2013, where it is marketed as Spedra^®^ by Menarini [7,8]. AVA offers advantages over its class-member drugs as it is more specific to phosphodiesterase type 5 receptors in comparison to the other PDE5 inhibitors and faster in onset, with an onset of action of 0.25 h post-administration [7]. However, AVA exhibits a poor pharmacokinetic profile, having a shorter half-life, poor water solubility (lipophilic drug), and higher protein binding level compared to the other PDE5 inhibitors, leading to a higher volume of distribution and extensive first-pass metabolism, hence a relatively lower bioavailability following oral administration [9,10]. In addition, AVA users may have the usual PDE5 inhibitors’ side effects, such as headache, flushing, or dizziness, and even with the higher specificity of AVA, a clinical study showed a sudden visual impairment, as with other PDE5 inhibitors [11]. AVA is insoluble in water (0.03 mg/mL), partially soluble in ethanol, with a 485 Dalton molecular weight, and a partition coefficient of 2.78, dissolvable in 0.10 mol/L HCl, and it exhibits the first-pass effect. Subsequently, the improvement of its bioavailability is vital for better clinical applications using an effective formulation [12]. These features frame AVA as a good candidate for nanoparticulate formulation and transdermal application.

Utilizing nanotechnology has been recently discussed and applied to various current conventional chemical drugs [13]. In other words, synthesizing current medicine at the level of the nano-scale delivery system, either by changing its size or shape, would benefit its physicochemical properties [14], essentially due to the increase in the surface-area-to-volume ratio of the newly formed material [15]. Several techniques of novel delivery systems, including nanoemulsions, nanovesicles, liposomes, and ethosomes, have recently been used to deliver AVA across different routes of administrations [16]. These novel systems exhibit superior pharmacological properties but with drawbacks.

Vitamin E d-α-tocopherol polyethylene glycol succinate (TPGS) is a safe and FDA-approved adjuvant that has well-established applications in delivery systems of drugs, particularly TPGS-based formulations and TPGS-based nanomedicines [17]. TPGS-based polymers can improve encapsulation efficiency, enhance solubility, facilitate permeation and intracellular uptake, and thus enhance the therapeutic effects of the formulated drug [18]. In addition, TPGS produces stable micelles in aqueous media owing to its low critical micelle concentration (0.02 *w*/*w*%), with a hydrophile/lipophile balance (HLB) value of 13.2 [19].

Transdermal delivery systems are a non-invasive, comfortable route for administrating drugs, with higher patient compliance rates [20]. Transdermal drug delivery systems also provide local drug absorption via the skin, closer to the site of action, which reduces drug-related side effects raised following oral delivery [20]. Several preparations exploit skin as a route of administration for the delivery of various active pharmaceutical ingredients. These transdermal applications range from the simple delivery of natural ingredients (such as curcumin and lycopene) for skincare and enhanced formulations for skin repair, to more advanced transdermal applications for the treatment of infections, inflammations, and local pain management [21]. Currently, in the market there are numerous successful products consisting of transdermal drug delivery formulations for indications of angina (NITRO-DUR^®^), depression (Emsam^®^), and even for hypertension (Catapres- TTS^®^) [22]. Surely, as pathological conditions treated by local and non-invasive means, one could expect to see an improvement in patient compliance for treatment and fewer adverse effects, as is the case in the treatment of skin cancer. Studies of skin cancer treatments show good antitumor effects and enhanced safety measures when using sophisticated highly functionalized forms of nanoparticles to enhance and target the antitumor drugs locally as a topical transdermal gel preparation [23,24,25]. These examples all prove the feasibility and great potential of transdermal drug delivery systems as an excellent alternative, ensuring compliance and an efficient route for drug administration.

Furthermore, transdermal drug delivery bypasses the enterohepatic circulation, thereby avoiding first-pass metabolism and thus enhancing the bioavailability of AVA [26]. Drugs with lipophilic properties showed better diffusion across the skin barrier of the stratum corneum, especially molecules that have low molecular weight (<600 Dalton) [27]. This ability to permeate through the stratum corneum is key in transdermal drug delivery as it is the main obstacle in this route of administration.

In this work, AVA was formulated for transdermal delivery applications, taking advantage of its lipophilic properties and relatively smaller molecular weight (485 Dalton), which makes AVA a good candidate for transdermal delivery through the skin’s stratum corneum. Nonetheless, combining the use of TPGS-based polymer with span 85 to form an efficient mixed micellar (MM) system of AVA for transdermal application is essential for the improvement of skin permeation and efficient treatments. The novel micelles structure of the AVA formulation was embodied in a transdermal film, providing dual delivery enhancements, aided by a Box–Behnken experimental design.

## 2. Materials and Methods

### 2.1. Materials

Avanafil (AVA) was supplied by Jinlan-Pharm-Drugs Technology Co., Ltd. (Hangzhou, China). D-α-Tocopherol polyethylene glycol 1000 succinate (TPGS), Sorbitan trioleate (Span 85), fluorescein isothiocyanate (FITC), eucalyptol 99%, propylene glycol, and ethanol were obtained from Sigma-Aldrich (St. Louis, MO, USA). Hydroxypropylmethylcellulose (HPMC), 4000 cp, was purchased from Spectrum Chemical Manufacturing Corporation (New Brunswick, NJ, USA). Methanol and acetonitrile were from Merck (Darmstadt, Germany). Ammonium acetate and phosphoric acid were obtained from Honeywell Riedel-de Haën AG (Wunstorfer, Seelze, Germany).

### 2.2. Formulation and Optimization of AVA-MM-Loaded Transdermal Film

Three factors and two responses, with a three-level Box–Behnken design (BBD), utilizing Statgraphics^®^ 18 Centurion Software (VA, USA) were employed in this study. Fifteen formulations were generated to optimize the AVA permeation from the transdermal film. The variables were the hydrophile-lipophile balance (HLB) of the surfactant (X_1_), the concentration of mixed micelles (MMs) in the film (X_2_), and eucalyptol (permeation enhancer) concentration (X_3_). The responses were the initial permeation percent after 1 h (Y_1_) and the cumulative permeation percent after 24 h (Y_2_) (Table 1). Based on the BBD, the software produced 15 randomized runs of independent variables, as seen in Table 2.

AVA-MM was prepared as previously reported, with modifications [28]. Briefly, AVA was added to a mixture of TPGS and span 85, according to the HLB value indicated by the design, dissolved in ethanol (15 mL), then poured into 20 mL distilled water under stirring conditions, and left on the stirrer for 4 h at ambient temperature. After that, ethanol was removed using a rotavapor. The volume of water was readjusted to 20 mL after ethanol evaporation.

The prepared AVA-MM particle size, utilized in the experimental design, was assessed utilizing a Zetasizer Nano ZSP (Malvern Panalytical Ltd. Malvern, UK). A sample of the AVA-MM solution was subjected to an appropriate dilution (1.5 mL) and then measured.

For the preparation of AVA transdermal films, the prepared AVA-MMs were dispersed in 50 mL distilled water, and then both HPMC (2% *w*/*v*) and propylene glycol (plasticizer, 2% *w*/*v*) were dispersed in the aqueous dispersion while stirring [29]. Eucalyptol (penetration enhancer) was added, in a concentration based on the proposed experimental design, and the formed gel was kept at 4 °C for 24 h. After that, the clear gel was poured into a Petri dish and dried at 40 °C. Transdermal film loaded with raw AVA was prepared by the same procedure, except AVA-MM was replaced with raw AVA.

### 2.3. Ex Vivo Permeation of AVA-Loaded Transdermal Film

The AVA ex vivo permeation study was conducted utilizing a USP dissolution apparatus (Pharma test, Hainburg, Germany) as previously described [30]. Freshly isolated rat skin (male Wistar rats (200–250 g weight) was used as a membrane. The isolated skin (3.14 cm^2^ effective surface area) was stretched on one end of the glass tube. The skin’s other side (interior surface) was touching the receptor medium (receptor compartment). A film sample with an area of one cm^2^ was put in the tube. The volume of phosphate-buffered solution receptor medium (pH 7) was 500 mL. The medium was kept at 37 °C ± 0.50 °C and 75 rpm stirring. At predetermined time points, withdrawn samples (3 mL) were replaced with fresh medium.

### 2.4. Prediction of the Optimized Formulation

The obtained data (15 formulae) were analyzed by the software, using analysis of variance (ANOVA), followed by the multiple response optimization. The optimum value for the three variables was determined.

### 2.5. Study of Optimized Transdermal Film Using a Fluorescence Laser Microscope

The transport of the formula through the skin was investigated utilizing a Zeiss Axio Observer D1 inverted DIC Fluorescence microscope (Carl Zeiss AG, Oberkochen, Germany). The filter used was 470/40 nm excitation, 495 beam splitter, and 525/50 nm emission. Images were acquired with identical acquisition parameters, with minimum excitation and gain. Optimized film loaded with FITC, instead of AVA, was prepared as reported before [19]. A raw FITC-loaded transdermal film was used as a control. The prepared films were mounted on skin patches and investigated using an automated Franz diffusion apparatus [30]. A skin patch of each cell was taken out after 0.50, 3, and 6 h, then fixed in formaldehyde solution, sectioned by a microtome from paraffin wax skin samples, and investigated under the microscope.

### 2.6. In Vivo Pharmacokinetic Studies

#### 2.6.1. Study Design

A single-dose one-period parallel design for the study was carried out. The animal experimental protocol was approved by the Research Ethics Committee, Faculty of Pharmacy, King Abdulaziz University (Approval No. PH-125-41). The study complied with the international guidelines for animal care.

#### 2.6.2. Animal Handling

Twenty-four male Wistar rats (200–250 g weight) were utilized in this investigation. General and environmental conditions were strictly monitored. Animals were distributed randomly into 2 groups (12/group). Group I (reference group, 12 animals divided into 2 sub-groups for blood sampling by alternative methods) received topically 20 mg/kg dose of raw (pure) AVA-loaded film. Group II (test group, 12 animals divided into 2 sub-groups as in group I) received topically 20 mg/kg dose of the optimized AVA-MM-loaded film. The film area (2.5 cm^2^) of uniform thickness was employed on the dorsal surface of each animal by adhesive tape.

#### 2.6.3. Blood Sampling

Blood samples (250 μL) were collected via the oculi choroidal vein at 0.50, 1.0, 2.0, 4.0, 8.0, 12.0, and 24.0 h after the transdermal dose under light ether anesthesia. To circumvent hypovolemia in rats as per the advice of the local ethical committee, each animal group was subdivided into two sub-groups and blood sampling points were distributed between the two subgroups alternatively. Animals were allowed free access to food and drinking water between time points of blood collection to avoid dehydration. Plasma samples were obtained at 2850× *g* for 5 min and frozen at −20 °C until an analysis was performed. AVA concentrations were measured using the HPLC method.

#### 2.6.4. Pharmacokinetics Parameters Evaluation

The optimized AVA-MM-loaded transdermal film pharmacokinetic parameters were evaluated in comparison with raw AVA-loaded film on male Wistar rats. Both formulations were applied topically as described in the animal handling section. The AVA concentration and the analysis of the parameters were calculated using the non-compartmental model, utilizing PKsolver [31]. AVA maximum plasma concentration (C_max_), AVA time point of C_max_ (T_max_), area under AVA curve from zero to the last measurable point (AUC _(0–24)_), area under the first moment plasma curve from time zero to infinity (AUMC_(0–inf)_), AVA terminal elimination rate constant (K_e_), AVA mean residence time (MRT_(0–inf)_), and AVA elimination half-life (t_1/2_) were calculated. The relative bioavailability of the optimized AVA-MM-loaded film compared with raw AVA film was determined.

#### 2.6.5. Chromatographic Conditions

AVA plasma sample concentrations were determined using a PerkinElmer ultraviolet spectroscopic detector equipped with a variable wavelength, adjusted at 230 nm, along with a quaternary pump, autosampler, vacuum degasser, and Winchrom software. Chromatographic separation was performed on a Phenomenex, RP Hi-Q-Sil C18, 250 mm × 4.6 mm, 5-μm column (Phenomenex, Torrance, CA) at room temperature. Acetonitrile, methanol, and a 0.05 M ammonium acetate buffer, pH 3 (30:20:50 *v*/*v*/*v*) was the phase that was pumped at a flow rate of 1.5 mL/min. For AVA extraction from plasma samples, 0.50 mL of a mixture of acetonitrile:methanol (1:1) was added, vortexed for 1 min, and centrifuged at 5000 rpm for 10 min, then 40 µL was injected into the HPLC system. The internal standard (100 ng/mL sildenafil solution in the mobile phase) was prepared by dissolving 10 mg, accurately weighed, of the compound in 100 mL of acetonitrile. The developed method was validated and deemed to be precise, accurate, sensitive, selective, and robust, with 2 and 0.50 ng/mL as the limits of quantitation and detections, respectively.

### 2.7. Statistical Analysis

All statistical analyses were executed using GraphPad Prism 8 software for Windows (San Diego, CA, USA). Regarding the plasma concentration-time curve, two-way ANOVA, followed by Sidak’s multiple comparisons test, was done to compare each means with the other at all time points and assess the significance between groups. Finally, a two-tailed unpaired *t*-test was used to assess the parameters of the formulations.

## 3. Results and Discussion

### 3.1. Optimization of AVA-MM-Loaded Transdermal Films

AVA-MM-loaded-film with an optimum permeation profile was optimized using BBD. The proposed formulations of tge prepared AVA-MM-loaded transdermal film, according to the experimental response surface design, showed a mixed micellar composition with a particle size range of 169.9 ± 16.3 nm (F11) to 291.70 ± 23.6 nm (F2) and with polydispersity index (PDI) values not exceeding 0.32 for the prepared AVA-MM (15 formulations), indicating adequately dispersed samples [32].

#### 3.1.1. Effect of Variables on the AVA Permeation (Y_1_ and Y_2_) Behavior

The determination of AVA permeation is required in order to assess AVA availability for its absorption at an adequate plasma level with the use of transdermal film. The assessment of the AVA initial and cumulative permeation from the studied films revealed variations ranging from 5.96% in F14 to 17.81% in F5 and from 18.86% in F9 to 43.95% in F11, respectively (Table 2). The generated polynomial equations for the investigated responses are presented in Equations (1) and (2).

(1) $$\begin{array}{ll} Initial\;AVA\;permeation\;\left(Y_{1}\right) & \\ & =11.625-2.449X_{1}-1.651X_{2}+1.363X_{3}+0.252X_{1}^{2}+0.004X_{1}X_{2}+0.338X_{1}X_{3}\\ & +0.323X_{2}^{2}-0.285X_{2}X_{3}-0.276X_{3}^{2} \end{array}$$

(2) $$\begin{array}{ll} Cumulative\;AVA\;permeation\;\left(Y_{2}\right) & \\ & =34.2634-8.578X_{1}+3.668X_{2}-2.388X_{3}+0.632X_{1}^{2}+0.162X_{1}X_{2}+1.125X_{1}X_{3}\\ & -0.279X_{2}^{2}-0.884X_{2}X_{3}+0.2875X_{3}^{2} \end{array}$$

**Figure 1:**
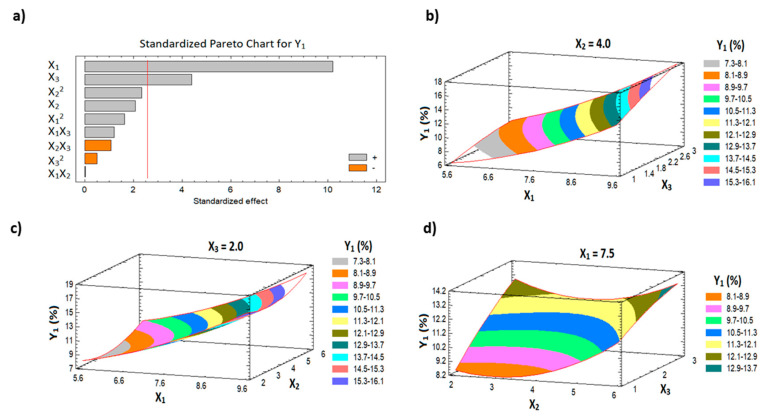
Pareto chart (**a**) and response surface plots (**b**–**d**) for Y_1_.

**Figure 2:**
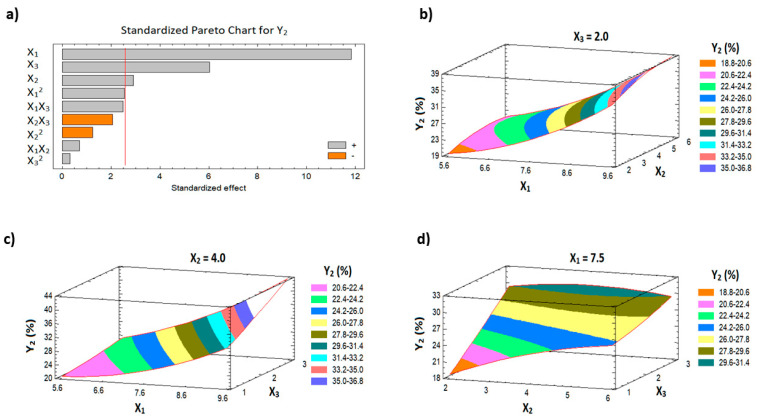
Pareto chart (**a**) and response surface plots (**b**–**d**) for Y_2_.

Table 3 shows the ANOVA statistical analysis for Y_1_ and Y_2_. In addition, Pareto charts are presented in Figure 1a and Figure 2a. The results indicate a significant positive effect of both the HLB of surfactant (X_1_) and the percentage of the penetration enhancer (X_3_) on the initial permeation of the drug from the film (Y_1_), with *p*-values of 0.0002 and 0.0071, respectively. With the same trend, all the studied factors have a significant positive effect on the cumulative AVA permeation (Y_2_) with *p*-values of 0.0001, 0.0336, and 0.0018 for X_1_, X_2_, and X_3_, respectively (Table 3). The 3D surface plots (Figure 1b–d and Figure 2b–d) illustrate the effects of the factors on the initial and the cumulative AVA permeation. Finally, the interaction and the quadratic terms between the factors showed no significant effect on both Y_1_ and Y_2_.

HLB value is an important criterion for the emulsification efficiency and entrapment of drugs. For the O/W system, as the HLB value increased the emulsification efficiency was improved and the entrapment of AVA (hydrophobic drug) within the core of the micelle was improved [33]. TPGS enhances drug transport across biological membrane barriers through its emulsifier activity, and this could be due to its P-glycoprotein (P-gp) inhibition [34,35]. Furthermore, TPGS-micelles demonstrated improved entrapment of drugs within the core of the micelles [17,36]. Furthermore, increased MM concentration in the film led to an increased percentage of the drug dissolved in the cores of the MMs.

The direct relationship between the percentage of penetration enhancer with both initial and cumulative AVA permeation is attributed to the penetration enhancer’s mechanism of action [37,38,39]. The mechanism of action is either by reversible disruption of the lipid bilayer in the stratum corneum or by modifying the solvent nature of the stratum corneum [37]. Another reported mechanism of the penetration enhancer is through interaction with the keratin structure in the corneocytes, which improves the drug diffusion coefficient [40]. Penetration enhancers can act by one or more of the indicated mechanisms [41].

#### 3.1.2. Prediction of the Optimized AVA-MM-Loaded Transdermal Film

**Figure 3:**
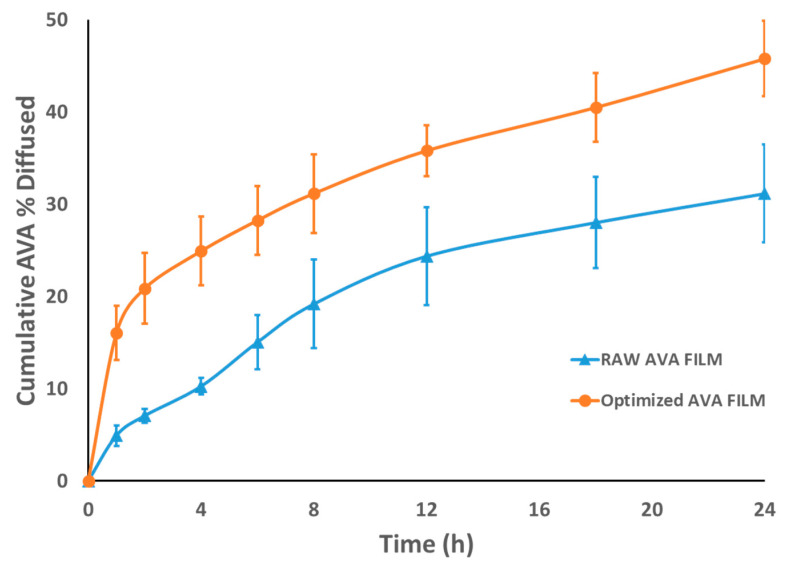
Ex vivo permeation profile of raw AVA-loaded film and optimized AVA-MM-loaded film. All data are expressed as the mean ± SD of three independent experiments.

The optimum level of the factors was predicted, after the data analysis, to be 9.4 for the HLB of the surfactant used, 5.12% for the percentage of MM, and 2.99% for the penetration enhancer, to achieve an optimum AVA permeation profile from the film. The level combinations maximized the desirability function over the indicated region to be 0.965. The optimized AVA-loaded transdermal film demonstrated an improved permeation pattern when compared to raw AVA-loaded film (Figure 3). The observed and predicted values of the optimized formula were very close, with an error percentage < 5% (Table 4).

### 3.2. Ex Vivo Fluorescence Microscope Investigation of the Optimized Film

**Figure 4:**
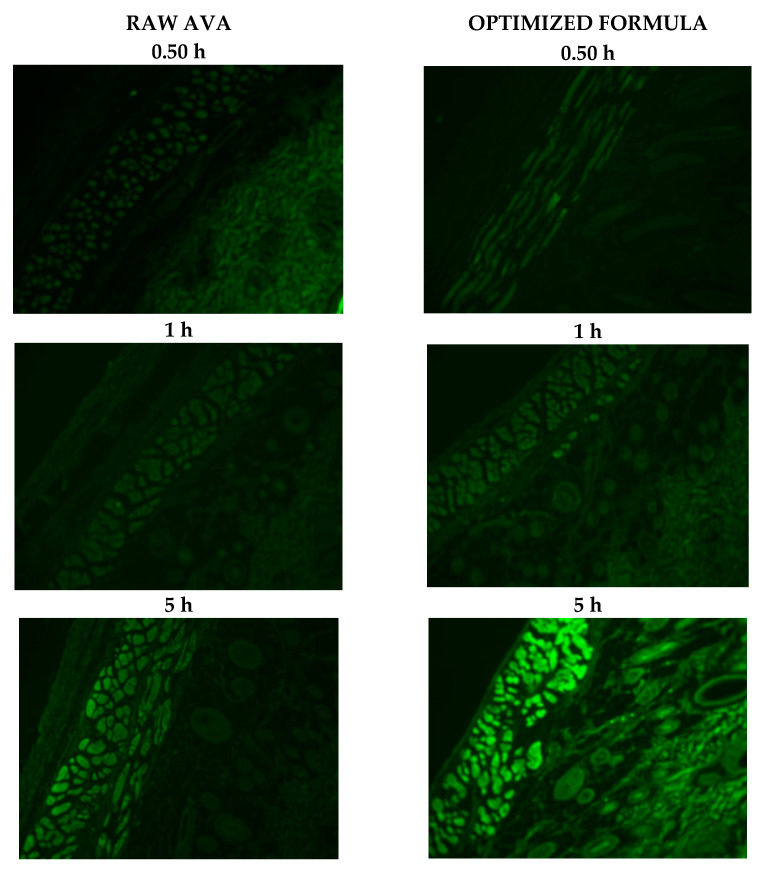
Fluorescence laser microscope images for transdermal application of raw FITC- and optimized formula FITC-loaded films after 0.50, 1, and 5 h (magnification 400× *g*).

The transport of raw FITC-loaded and optimized FITC-loaded films are displayed in Figure 4. It has to be mentioned that the loading of FITC into the MMs did not affect the characters of the MMs in terms of particle size. The FITC-loaded optimized film transported the payload across the skin layers. This was verified by the level of fluorescence intensity. On the other hand, the raw FITC film showed lower intensity when compared with the optimized film. This finding indicates that the optimized film delivered the payload to deeper skin layers effectively. This finding is in agreement with previous investigations that revealed the ability of nanocarriers to improve skin permeation for loaded drugs [19,33]. The distribution of AVA at the nano-scale of the prepared AVA-MM is responsible for the enhancement of the penetration ability as a result of the increased surface area, which improves its interaction with the stratum corneum cells, which results in improved AVA permeation [29]. According to these results, the optimized MM formulation shows great potential for improved AVA bioavailability.

### 3.3. In Vivo Pharmacokinetic Evaluation

**Figure 5:**
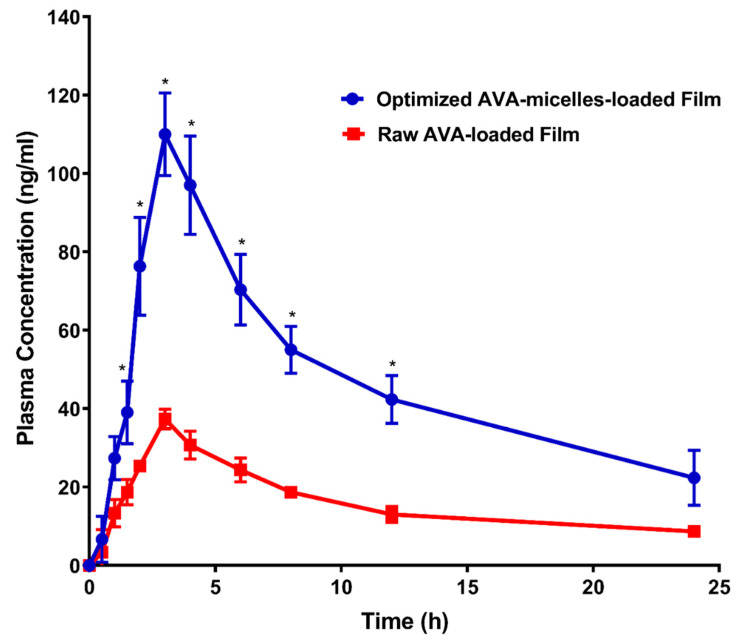
Avanafil plasma time concentration curves after transdermal application of raw AVA-loaded film and optimized AVA-MM-loaded film. Data represent the mean value ± standard deviation (SD). * Significant at *p* < 0.05.

The pharmacokinetic investigation of the optimized AVA-loaded transdermal film, following topical application to male Wistar rats, was studied and compared with raw AVA film as a reference. The AVA plasma concentrations of the optimized AVA-MM-loaded transdermal film and the raw AVA-loaded films are displayed in Figure 5. Furthermore, Table 5 demonstrates the pharmacokinetic parameters. The optimized AVA-MM-loaded transdermal film indicated a significantly (*p* < 0.05) higher AVA Cmax (approximately 3-fold) and AUC when compared with the raw AVA-loaded film (Table 5). The optimized AVA-MM-loaded transdermal film exhibited higher relative bioavailability when compared with the raw AVA-loaded film. The maximum concentration of both investigated formulations was reached after 3 h. No significant (*p* < 0.05) difference in elimination half-life or t1/2 was detected between the two films.

AVA-MM-loaded transdermal film showed a relative bioavailability of 289.5% in comparison with the raw AVA-loaded film. This result indicates that the development of AVA-MM-loaded transdermal film enhanced the transdermal permeation of the drug. As indicated in the earlier sections, TPGS MM enhances AVA transport across biological membrane barriers and modifies the biological response by inhibiting P-gp [34,35]. This finding indicates that formulating AVA with TPGS MMs as a transdermal film shows great promise in the delivery of AVA across skin layers. Furthermore, more studies are required to clarify the mechanism of increased transdermal delivery from MM film.

## 4. Conclusions

The results indicated the success of the Box–Behnken experimental design in optimizing the permeation pattern of AVA using transdermal film. The optimized AVA-MM-loaded transdermal film deduced by the Box–Behnken design demonstrated AVA permeation with an enhanced pattern in comparison with raw AVA-loaded film. The AVA-MM-loaded film was able to deliver the payload to deeper skin layers when compared to raw AVA-loaded film. The optimized AVA-TPGS MM-loaded film exhibited improved pharmacokinetic parameters, with improved bioavailability in comparison to the raw AVA-loaded film. This finding confirms that Avanafil-TPGS mixed micelle-loaded film is a promising transdermal delivery system for AVA.

## Figures and Tables

**Table 1:** Box–Behnken design attributes, involving factors and their selected levels with responses and their constraints and goals.

Factor	Low	High	Units
X_1_: HLB of surfactant	5.60	9.40	
X_2_: Concentration of MMs in the film	0.20	2.00	%
X_3_: The percentage of penetration enhancer in the film	1.00	3.00	%
**Response**	**Low**	**High**	**Goal**
Y_1_: Initial permeation after 1 h (%)	5.96	17.81	Maximize
Y_2_: Cumulative permeation after 24 h (%)	18.86	43.95	Maximize

**Table 2:** Experimental matrix of AVA-MM-loaded transdermal film as suggested by the Box–Behnken design, with the observed and the fitted values of the responses (Y_1_ and Y_2_).

Run	HLB of Surfactant	Conc of MM in the Film	Penetration Enhancer	Initial Permeation (%)		Cumulative Permeation (%)	
				Observed Value	Fitted Value	Observed Value	Fitted Value
1	7.50	4.00	2.00	10.40	10.51	26.46	26.70
2	9.40	4.00	1.00	11.55	12.68	30.91	30.67
3	5.60	4.00	3.00	9.45	8.32	23.35	23.59
4	5.60	6.00	2.00	9.31	9.64	22.24	21.81
5	9.40	6.00	2.00	17.81	17.34	35.97	37.46
6	7.50	2.00	3.00	12.3	12.97	28.29	29.54
7	7.50	6.00	1.00	11.89	11.22	26.99	25.74
8	7.50	4.00	2.00	10.44	10.51	26.09	26.70
9	7.50	2.00	1.00	9.32	8.52	18.86	18.67
10	5.60	2.00	2.00	7.64	8.11	20.99	19.51
11	9.40	4.00	3.00	17.61	17.28	43.95	42.28
12	9.40	2.00	2.00	16.08	15.75	32.26	32.69
13	7.50	6.00	3.00	12.59	13.39	29.35	29.54
14	5.60	4.00	1.00	5.96	6.30	18.86	20.53
15	7.50	4.00	2.00	10.69	10.51	27.55	26.70

**Note:** The observed values of Y_1_ and Y_2_ represent the means of three determinations; standard deviations were <5% of the mean and thus are omitted from the table.

**Table 3:** Statistical analysis of variance (ANOVA) of the responses (Y_1_ and Y_2_) results.

Factors	Initial Permeation (Y_1_), %			Cumulative Permeation (Y_2_), %		
	Estimate	F-Ratio	P-Value	Estimate	F-Ratio	P-Value
X_1_	7.6725	103.9100	0.0002 *	14.4125	140.14	0.0001 *
X_2_	1.5650	4.3200	0.0921	3.5375	8.4400	0.0336 *
X_3_	3.3075	19.3100	0.0071 *	7.3300	36.2500	0.0018 *
X_1_ X_1_	1.8175	2.6900	0.1618	4.5600	6.4700	0.0516
X_1_ X_2_	0.0300	0.0000	0.9786	1.2300	0.5100	0.5069
X_1_ X_3_	1.2850	1.4600	0.2813	4.2750	6.1600	0.0556
X_2_ X_2_	2.5825	5.4300	0.0671	−2.2300	1.5500	0.2685
X_2_ X_3_	−1.1400	1.1500	0.3331	−3.5350	4.2200	0.0953
X_3_ X_3_	−0.5525	0.2500	0.6392	0.5750	0.1000	0.7613
R^2^	96.51			97.61		
Adj. R^2^	90.23			93.31		
SE	1.06			1.72		
MAE	0.52			0.81		

**Note:** * Significant effect of factors on individual responses. Abbreviations: X_1_, the HLB of surfactant; X_2_, the concentration of MM in the film; X_3_, the percentage of penetration enhancer; X_1_X_2_, X_1_X_3_, X_2_X_3_, the interaction term between the factors; X_1_X_1_, X_2_X_2_, and X_3_X_3_, the quadratic terms between the factors; R^2^, R-squared; Adj-R^2^, adjusted R-squared; SEE, standard error of estimate; and MAE, mean absolute error.

**Table 4:** Composition of the optimized AVA-MM-loaded transdermal film with the observed, fitted, and residual values.

Factor	Optimum	Response	Observed Value	Fitted Value	Residual
X_1_: the HLB of surfactant	9.40	Y_1_: Initial permeation (%)	16.21	17.81	1.60
X_2_: the percentage of MMs in the film	5.12				
X_3_: the percentage of penetration enhancer in the film	2.99	Y_2_: Cumulative permeation (%)	43.95	42.26	1.69

**Table 5:** Pharmacokinetic parameters after transdermal application of a single dose (20 mg/kg) of the optimized AVA-MM-loaded transdermal film compared with raw avanafil-loaded film.

Parameter	Unit	Optimized AVA-TPGS MM-Loaded FIlm	Raw AVA-Loaded Film
		Average ± SD	Average ± SD
K_e_	1/h	0.06 ± 0.02	0.06 ± 0.01
t_1/2_	h	13.04 ± 4.84	12.73 ± 3.58
T_max_	h	3.0 ± 0.0	3.0 ± 0.0
C_max_	ng/ml	110.0 ± 10.54 *	37.33 ± 2.52
AUC _0–24_	ng/mL*h	1129.417 ± 155.54 *	382.58 ± 40.06
AUC _0–inf_	ng/mL*h	1579.78 ± 391.17 *	545.68 ± 108.95
AUMC _0–inf_	ng/mL*h^2^	31,162.81 ± 17,693.69	10,740.85 ± 4486.28
MRT _0–inf_	h	18.77 ± 6.50	19.15 ± 4.09

**Note:** * Significant difference at *p* < 0.05 using a two-tailed unpaired *t*-test.
